# The Role of Diet during Pregnancy in Protecting against Gestational Diabetes Mellitus in a Population with Mediterranean Dietary Habits: A Cross-Sectional Study

**DOI:** 10.3390/jcm12051857

**Published:** 2023-02-26

**Authors:** Ermioni Tsarna, Anna Eleftheriades, Efthymia Tsomi, Georgia Ziogou, Panagiotis Vakas, Theodoros Panoskaltsis, Panagiotis Christopoulos

**Affiliations:** 1 2nd Department of Obstetrics and Gynecology, Aretaieion University Hospital, Athens Medical School, 11527 Athens, Greece; 2 Postgraduate Programme “Maternal Fetal Medicine”, Medical School, National and Kapodistrian University of Athens, 11527 Athens, Greece

**Keywords:** gestational diabetes mellitus, diet, pregnancy, GDM

## Abstract

Gestational diabetes mellitus (GDM) is a common metabolic disorder among pregnant women. Dietary habits during pregnancy might alter the risk of GDM development, and populations following the Mediterranean diet are relatively understudied. This was a cross-sectional, observational study of 193 low-risk women admitted to a private maternity hospital in Greece to give birth. Food frequency data on specific food categories, selected based on previous research, were analyzed. Logistic regression models, both crude and adjusted for maternal age, body mass index before pregnancy, and gestational weight gain, were fitted. We observed no association of carbohydrate-rich meals, sweets, soft drinks, coffee, rice, pasta, bread and crackers, potatoes, lentils, and juices with GDM diagnosis. Cereals (crude *p* = 0.045, adjusted *p* = 0.095) and fruits and vegetables (crude *p* = 0.07, adjusted *p* = 0.04) appeared to have a protective effect against GDM, while frequent tea consumption was linked to higher risk of GDM development (crude *p* = 0.067, adjusted *p* = 0.035). These results strengthen previously identified associations and underline the importance and potential impact of changing dietary habits even during pregnancy in adjusting one’s risk of metabolic pregnancy complications, such as GDM. The importance of healthy dietary habits is highlighted, with the goal of raising awareness amongst obstetric care specialists regarding the provision of systematic nutrition recommendations to pregnant women.

## 1. Introduction

Gestational diabetes mellitus (GDM) is a common metabolic disorder diagnosed during pregnancy in women who were normoglycemic before conception [1,2]. According to the International Association of Diabetes and Pregnancy Study Groups (IADPSG), the clinical diagnosis of GDM requires at least one blood glucose value above the normal limit for pregnancy in the 75 g oral glucose tolerance test (OGTT), performed at 24–28 weeks of gestation [3]. GDM has been associated in large international cohorts with several unfavorable pregnancy and perinatal outcomes, including preeclampsia, higher rates of caesarean section and preterm birth, macrosomia and shoulder dystocia, neonatal hypoglycemia and higher insulin levels, neonatal jaundice, and higher rates of neonatal intensive care unit admission [3,4]. The aforementioned outcomes are positively associated with blood glucose levels during pregnancy, even among normoglycemic women [3,5]. Lastly, women diagnosed with GDM have a greater risk of developing type II diabetes mellitus later in their lives [3,6].

The role of diet during pregnancy in GDM development has been explored in many observational epidemiological studies and randomized controlled trials (RCTs). In a relevant systematic review, 25 dietary observational studies were identified [7]. Higher total dietary fiber consumption, fruit and cereal consumption, caffeine intake, and tea consumption were linked to a lower risk of developing GDM [7]. On the contrary, the consumption of sugar-sweetened beverages and potatoes was associated with a greater risk of GDM [7]. It is worth mentioning that among these 25 studies, only two included populations that followed a Mediterranean diet, and in fact, one of these did not examine food intake but rather adherence to the Mediterranean diet in relation to GDM diagnosis [8]. With regard to evidence from RCTs regarding the role of dietary habits in GDM risk, a relevant Cochrane review of five RCTs involving 1279 pregnant women concluded that dietary advice interventions during pregnancy may be effective for GDM prevention, although the meta-analysis results were not statistically significant (RR = 0.60, 0.35–1.04 95% CI) [9]. However, the quality of evidence was very low and none of these studies were carried out in a Mediterranean diet population [9]. Nutritional epidemiology data are prone to confounding bias that is specific to the dietary habits of the studied population. Inherently, a greater consumption of one food category could be associated with a lower consumption of others, higher total calorie intake, or a combination of the above. Thus, a protective effect of a specific food category might actually reflect the protective effect of lower total calorie intake or the harmful effect of other food categories. Therefore, it is important to confirm results from nutritional epidemiological studies and RCTs in populations following different dietary habits.

Although there is no single definition, the Mediterranean diet is characterized by being low in saturated fat and high in vegetable oils, and was firstly observed in Greece and Southern Italy during the 1960s [10]. The Mediterranean diet typically contains a high amount of fruit, vegetables, seeds, nuts, and whole grains, as well as olive oil as the main source of monounsaturated fat. It also includes a moderate amount of fish, poultry, and dairy [10]. The most important characteristic of this dietary pattern is that it has been associated with a reduced risk of coronary heart disease compared to northern European countries and the United States, and interestingly enough, numerous studies report that this happens in a dose-dependent fashion [10]. In other observational studies, the Mediterranean diet has been associated with a decreased incidence of Parkinson’s and Alzheimer’s diseases, and different types of cancers, including colorectal, prostate, oropharyngeal, and breast cancers [10]. As far as pregnancy is concerned, a recent systematic review by Zaragoza et al. published in 2022 concluded that the Mediterranean diet is an optimal diet to consume during pregnancy, since it provides an adequate supply of micronutrients while controlling weight gain during gestation [11]. Growing evidence also suggests the beneficial effect of the Mediterranean diet during pregnancy on children’s health, increasing small airway function in childhood and having a protective role against asthma, allergy, and increased body weight [12,13]. Even though the Mediterranean diet has been shown to be beneficial during pregnancy, the role of specific food categories within the Mediterranean diet is not well understood, which poses a challenge when practitioners provide dietary advice for pregnant women who follow Mediterranean diet.

The effect of diet during pregnancy on GDM development in populations with the Mediterranean diet is relatively understudied. The aim of this study is to explore the role of dietary habits such as carbohydrate and caffeine consumption in the development of GDM, studying a population of low-risk pregnant women in Greece.

## 2. Materials and Methods

This was a cross-sectional observational study of consecutive pregnant women who were admitted to give birth from March 2019 until August 2019 in a private maternity hospital in Athens, Greece. Women with an elective caesarean section were excluded in order to select a low-risk population that would not be affected by other pregnancy-specific pathologic conditions, such as pregnancy-related hypertension and preeclampsia. In addition, women who did not speak Greek fluently were excluded from this study. After informed consent was obtained, a questionnaire was administered to the pregnant woman, while birth outcomes were recorded later by the research midwife. The study protocol was approved by the Research Ethics Committee of the Maternity Hospital (protocol number: 1516009106/27-11-2015).

Dietary habits during pregnancy were assessed with a semi-quantitative food frequency questionnaire in Greek [14]; the questions used in the statistical analysis of this study are presented in detail in Tables S1 and S2. Women were explicitly instructed to answer the dietary questionnaire taking into consideration the whole pregnancy period, rather than the third trimester. For the aim of this study, we used semi-quantitative data on the consumption frequency of fruits and vegetables, carbohydrate-rich meals, sweets, soft drinks, and coffee, and qualitative data on the consumption frequency of cereals, rice, pasta, bread and crackers, potatoes, lentils, tea, and juices. Unfortunately, a definition for cereals was not provided to the pregnant women. Therefore, apart from healthy choices, such as whole grain and unprocessed cereals, women might have also answered regarding unhealthy choices, such as processed and sugar-sweetened cereals. Furthermore, participants were asked about gravidity and parity status, GDM diagnosis based on the 75 g OGTT at 24–28 weeks of gestation and the IADPSG criteria for GDM diagnosis, pregnancy-related hypertension and preeclampsia, demographic characteristics, and anthropometric characteristics before and at the end of pregnancy [3]. The research midwife cross-checked the self-reported anthropometric characteristics with the ones recorded by the obstetrician who had followed up the pregnancy in the women’s medical folders, in order to ensure high accuracy of variables that were used in the statistical analysis.

For descriptive statistics of baseline characteristics of participants and food frequency data, we used means and standard deviation for continuous variables, and frequencies and proportions for categorical variables. Baseline characteristics between study participants with and without GDM were compared with the non-parametric Mann–Whitney U test for continuous variables, and chi-squared test for categorical variables. In order to examine the association of food frequency data with GDM diagnosis, crude and adjusted logistic regression models were fitted. The exposure variable was a categorical variable in all models, and the likelihood ratio test was used to calculate *p* values. The potential confounders used as covariates in the adjusted models were maternal age, body mass index (BMI) before pregnancy, and gestational weight gain. The last two were used as proxies for total energy intake, for which we did not have any direct data. The level of significance was 0.05 for all the aforementioned statistical analyses. It should be noted that there were no missing data among the variables used in the logistic regression models.

All statistical analyses were performed using R statistical software (version 4.1.2; R Foundation for Statistical Computing, Vienna, Austria) [15] and the software packages “tableone” [16] and “psych” [17]. The computer code used for the statistical analysis using the R statistical software can be provided upon request. For the reporting of this study’s results, “Strengthening the Reporting of Observational Studies in Epidemiology” (STROBE) guidelines have been followed [18].

## 3. Results

Out of 224 pregnant women invited to participate in this study, 193 provided informed consent and are included in this analysis. Mean maternal age at neonate’s birth was 31.5 years (Table 1). Pre-gestational BMI was on average 22.81, while gestational weight gain was 13.46 kg (Table 1). Sixty-five women (33.7%) gained weight within the Institute of Medicine (IOM) target range, 61 (31.6%) below the IOM target range, and 67 (34.7%) above the IOM target range (Table 1) [19]. The majority of participants, 124 (64.2%) women, were pregnant for the first time, 43 (22.3%) were pregnant for the second time, and 26 (13.5%) were pregnant for at least the third time (Table 1). With regard to smoking status, 85 (44%) were smoking before conception, 23 (11.9%) continued smoking during pregnancy, and 88 (45.6%) reported being exposed to passive smoking at home during pregnancy. Twelve women (6.2%) were diagnosed with GDM, 7 (3.6%) with pregnancy-induced hypertension, and no women were diagnosed with preeclampsia (Table 1). Concerning neonatal characteristics, mean gestational age at birth was 38.5 weeks, mean birthweight was 3075 g, and 88 (52.4%) of the neonates were assigned female sex at birth.

As far as dietary habits are concerned, during pregnancy, most women consumed meals that were rich in carbohydrates one to three times per day. In fact, 53 (27.5%) women consumed such a meal once daily, 86 (44.6%) twice daily, and 41 (21.2%) three times daily (Table S1). Fruits and vegetables were frequently chosen during pregnancy; once per day by 33 (17.1%) women, twice by 74 (38.3%), three times per day by 45 (23.3%), while 38 (19.7%) women ate fruits and vegetables four to five times per day (Table S1). As for sweets, 66 (34.2%) women consumed sweets once or twice weekly, but an alarming 28% (54 women) ate sweets every other day and 18.7% (36 women) did so daily (Table S1). The majority of women did not consume soft drinks during pregnancy (136 women or 70.8%) and only 23 (11.9%) consumed them on a daily basis (Table S1). With regard to coffee consumption, 39.9% (77 women) refrained from coffee during pregnancy, 14% (27 women) drank coffee on average three times per week, 39.4% (76 women) once daily, and 6.7% (13 women) drank two or more cups of coffee every day (Table S1). Regarding qualitative food frequency data, cereals were frequently consumed by 133 (68.9%) women, rice by 134 (69.4%), pasta by 158 (81.9%), bread and crackers by 129 (66.8%), potatoes by 137 (71%), lentils by 131 (67.9%), tea by 39 (20.2%), and juices by 140 (72.5%) women (Table S1).

In the analysis of the semi-quantitative food frequency data, the consumption frequency of carbohydrate-rich meals, sweets, soft drinks, and coffee did not correlate with GDM diagnosis, neither in the crude nor in the adjusted logistic regression models (Table 2). In contrast, fruit and vegetable consumption frequency was non-significant in the crude analysis (*p* = 0.07), but gained statistical significance in the adjusted analysis (*p* = 0.04) (Table 2). Since the null hypothesis is double-sided in the statistical logistic regression models, the odds ratios of each level of the categorical variable used for fruit and vegetable consumption frequency were checked, in order to decide if this significant association reflected a protective or harmful effect. Both in the crude and the adjusted logistic regression models, all odds ratios were below unity with no consumption being the reference category, indicating a protective role of fruit and vegetable consumption during pregnancy against GDM (Table S2). Naturally, the power for each level of fruit and vegetable consumption frequency variable was much lower than the power for the categorical variable as a whole, and none of these odds ratios reached statistical significance on each one.

In the analysis of the qualitative food frequency data, the frequent consumption of rice, pasta, bread and crackers, potatoes, lentils, and juices did not correlate with GDM diagnosis, neither in the crude nor in the adjusted logistic regression models (Table 3). Nonetheless, cereal consumption appeared to be protective against GDM in the crude logistic regression model (odds ratio 0.30, *p* = 0.045) (Table 3). However, this result lost its statistical significance once adjusting for maternal age, BMI before pregnancy, and gestational weight gain (odds ratio 0.35, *p* = 0.095) (Table 3). In contrast to cereal consumption, tea consumption appeared to be a risk factor for GDM diagnosis; this result was non-significant in the crude analysis (odds ratio 3.09, *p* = 0.067), but gained statistical significance in the adjusted analysis (odds ratio 3.97, *p* = 0.035) (Table 3).

## 4. Discussion

In this study of 193 pregnant women sampled from a low-risk population that follows the Mediterranean diet, consuming fruits, vegetables, and cereals during pregnancy appeared to decrease the risk of GDM diagnosis. In contrast, frequent tea consumption during pregnancy was associated with a higher risk of being diagnosed with GDM.

Fruits, vegetables, and cereals, which were shown to be protective against GDM in our study, are all well-known sources of fiber, in addition to having a low glycemic index. Several mechanisms have been proposed to explain why a high-fiber diet might ameliorate glucose metabolism. Gastric emptying is delayed in presence of high-fiber stomach content and digestion is slower in total, resulting in reduced glucose absorption [20]. In addition, a high-fiber diet has been linked to reduced appetite and total energy intake, and, consequently, reduced adiposity, which is a well-known risk factor for GDM as it enhances insulin intolerance [20]. It is not surprising, therefore, that our results are in line with previous research that has indicated the protective role of fruits, vegetables, and cereals against GDM, even though the vast majority of these research data did not originate from populations that follow a Mediterranean diet [7,9]. Thus, this protective effect seems to be robust across populations with different dietary habits.

The role of dietary carbohydrates in GDM development is quite complex. Counterintuitively, a low-carbohydrate dietary pattern has been associated with a higher risk of GDM [21,22]. Women who consume fewer carbohydrates on average consume more fat, which has been positively correlated with GDM risk [21,23]. Nonetheless, in our study, we did not observe any association of self-reported consumption frequency of carbohydrate-rich meals during pregnancy and GDM. With regard to the type of carbohydrates consumed, simple carbohydrates, as opposed to complex ones, are regarded as a contributing factor for GDM due to the steeper increase in blood glucose levels postprandially. It should be noted that the amount of fiber is also of importance, and even foods rich in complex carbohydrates might contribute to GDM if fiber content is low [23]. This might explain why refined grains and starches are considered as potentially harmful for GDM development [23]. In our study, sweets and soft drinks, which are rich in simple carbohydrates, rice, pasta, bread, and crackers, which are usually made from white starches, and potatoes, which have been shown to contribute to GDM development in other observational studies, did not correlate with GDM [7]. Similarly, lentils, which are rich in complex carbohydrates and fiber, as well as juices, which are rich in fiber even though they contain simple carbohydrates, did not correlate with GDM. Whether this lack of association is the result of low power or reflects a true lack of association in a population that follows a Mediterranean diet remains unclear.

Caffeine intake has been negatively correlated with GDM diagnosis, indicating a potentially protective role of caffeine [7,24]. Coffee phytochemicals may support the preservation of pancreatic beta cell function by preventing pancreatic cell damage during periods of high insulin secretion and the formation of cell-toxic amyloids [25]. Other theories for the beneficial effects of coffee consumption support the suggestion that coffee compounds lead to the activation of AMPK, which works as a switch between anabolism (ATP expenditure) and catabolism (ATP production) [26]. In contrast to the evidence supporting the suggestion that caffeine consumption may improve glucose metabolism, a few studies have suggested that in individuals with previously diagnosed diabetes, caffeine may have a negative impact on blood glucose concentration. A systematic review of randomized control trials published in 2013 that included a total of nine trials concluded that the consumption of moderate to high doses of a single daily caffeine supplement is associated with elevated post-prandial blood glucose concentrations [27]. These nine trials involved human participants who had the diagnosis of diabetes type I, type II, or GDM [27]. However, the underlying mechanism is not clearly understood and, as the authors imply, high coffee consumption may constitute a marker for other risk factors [27,28]. This systematic review did include a single study of patients with GDM [27]. This study by Robinson et al., which included women with and without GDM (control group), concluded that in the control group, caffeine did not significantly affect blood glucose, insulin sensitivity, or C-peptide, whereas in the GDM group, C-peptide was greater (*p* < 0.05), and the insulin sensitivity index was lower (*p* < 0.05) [27,29]. However, more studies and additional research are needed since the sample size was very small (women with GDM n = 8). In our study, neither coffee nor tea, both of which contain caffeine, appeared to have any protective effect against GDM [30]. It should be noted that among our study population, almost 40% of women refrained from coffee during pregnancy. Although the rationale for this refrainment was not recorded in our study, we suspect that it relates to concerns regarding the association of high caffeine consumption with pregnancy loss and fetal growth restriction [31]. In contrast, the frequent consumption of tea during pregnancy was linked to a higher risk of being diagnosed with GDM. Tea has been shown to protect against type II diabetes and its content of catechins and polyphenols that exert an antioxidant effect has been implicated as a possible biological explanation not related to caffeine per se [32,33]. Nonetheless, a previously published analysis of the association between tea consumption and GDM among 86,453 pregnancies from Denmark did not find a statistically significant association despite the large sample size, even though the effect estimates did indicate a potential protective effect of tea [34]. Unfortunately, in our study questionnaire, we did not include any questions on the type of tea consumed and whether it was consumed unsweetened, with honey, or with sugar. It should be noted that not only the type of tea but also brewing time have been shown to affect the levels of caffeine content, which makes it difficult to interpret tea consumption data in terms of caffeine intake [30]. Taking all the aforementioned into consideration, we cannot conclude if our finding of a positive association between tea consumption and GDM risk reflects a true association, an association of sugar consumption with GDM, or if it is merely a reflection of multiple testing.

A nutritious diet, balanced with regard to macronutrients and micronutrients, is considered important during gestation both for the mother and the child. In particular, balanced nutrition is thought to contribute to optimal fetal growth, is associated with a lower risk of unfavorable obstetrical and neonatal outcomes, and can affect maternal and child health long-term [35]. Notably, the latest research has suggested that the Mediterranean diet might contribute to improving the immune system of pregnant individuals, also strengthening their immune response to viral infections such as COVID-19 [36]. In contrast, inappropriate maternal nutrition, both in terms of under-nutrition and over-nutrition, has been linked with abnormal fetal growth patterns, including both small and large- for gestational age fetuses, respectively, and also with a higher long-term risk of developing obesity, nonalcoholic fatty liver disease (NAFLD), and cardiovascular diseases [35]. Dietary interventions during pregnancy have been researched and shown to be effective in the prevention of pregnancy-associated hypertension and reducing excessive weight gain during pregnancy [9]. Fibers, whole grains, nuts, vegetables and fruits, legumes, fish, and meals rich in monounsaturated fats are regarded as important components of a nutritious diet during pregnancy [35]. The importance of fatty acid consumption during pregnancy has been highlighted by an RCT, which reported that the consumption of mono- and unsaturated fatty acids from olive oil and pistachios may reduce the incidence of GDM [37]. In contrast, the consumption of limited amounts of refined grains, simple sugars, fatty red meat, processed foods, and trans- and saturated fats is advised during pregnancy [35]. Diets that consistently restrict a macronutrient have been proven to be ineffective and are not recommended for pregnant women, as they can result in micronutrient deficiency and may negatively impact child health [38]. In particular, carbohydrate-restricting diets during pregnancy have been associated with a higher incidence of neural tube defects and excessive weight gain during childhood [35].

To our knowledge, this is one of the very few studies to examine dietary habits during pregnancy and GDM risk in populations following a Mediterranean diet and, thus, it strengthens the robustness of previously found associations. The Mediterranean diet has been shown to be protective both against GDM development and type II diabetes mellitus development among women previously diagnosed with GDM. Nonetheless, the role of specific food categories within the Mediterranean diet remains unclear. In fact, a previous study by Karamanos et al., which was published in 2014 and included a total of 1076 pregnant women, concluded that the adherence to the Mediterranean diet during pregnancy can lead to a better glucose tolerance and, therefore, act protectively against GDM [8]. Interestingly, Tobias et al. demonstrated that the adherence to a healthy dietary pattern, such as the Mediterranean diet, could be associated with a lower type II diabetes mellitus risk among women who were previously diagnosed with GDM [39]. Another study from Tobias et al., published in 2012, which included 15,254 participants, showed that pre-pregnancy adherence to the Mediterranean diet was associated with a significant decrease in GDM risk [40]. These findings demonstrate the importance of the adherence to healthy dietary habits before, during, and after pregnancy, which can lead to a reduction in unfavourable perinatal outcomes as well as to a reduction in cardiovascular mortality.

Notwithstanding our findings, this study had several limitations. The sample size was quite small, and a low-risk population was sampled, resulting in a low incidence of GDM and, therefore, low power of this study. Consequently, we chose not to explore novel associations of GDM and dietary habits, which would have given rise to the issue of multiple testing as well, but rather selected specific dietary habits that have been previously recognized to be associated with the risk of GDM.

In addition, our study population may not be representative of the general Greek population. With regard to representativeness concerns, we observed that an alarming percentage of women (44%) smoked before getting pregnant, 11.9% continued smoking during pregnancy, and 45.6% of women were exposed to passive smoking during their pregnancy. Since smoking seemed alarmingly frequent in our study population and it is not known whether the effect of dietary habits on GDM risk varies between smokers and non-smokers, we compared smoking status in our study with other birth cohorts in studies that have been recently conducted in populations following the Mediterranean diet. the RHEA cohort, a population-based birth cohort from the Heraklion region in Greece that recruited women in 2007 and 2008, 43.7% of women smoked before pregnancy and 23.8% continued smoking during their pregnancy [41]. In INMA, a birth cohort from Spain recruiting women between 2003 and 2008, 65.8% of pregnant women were exposed to passive smoking at home and 32.5% of them were actively smoking [42]. In contrast, in NASCITA, a representative birth cohort from Italy that recruited women in 2019 and 2020, only 6.5% of women continued smoking during pregnancy [43]. Therefore, in our study population, smoking during pregnancy was less frequent than in other Greek and Spanish birth cohorts, but more frequent than in Italian cohorts. Even though smoking status during pregnancy does not seem to raise a concern regarding sample representativeness, our sample might still differ from the general population with regard to socioeconomic position, occupational activities, and environmental exposures during pregnancy. Since our study participants were recruited in a private maternity hospital in the capital city of Greece, women of lower socioeconomic status are expected to be underrepresented. The same holds true for women with agricultural and livestock occupations that are more common in the Greek countryside, and involve high levels of physical activity, which might interact with dietary habits. Similarly, environmental exposures are expected to differ significantly between urban populations, as in our study, and rural populations. The aforementioned might influence the relevance of our results for the women who are not well represented in our study, but does not affect the scientific inference of our results, since the representativeness of the sample does not bias the results of research on potentially causal associations [44].

In addition, the data collection was cross-sectional; recall bias might have affected our food frequency data and first trimester dietary habits might not be well reflected, especially if pregnant women changed their dietary habits as pregnancy progressed. According to usual care in Greece, women who are diagnosed with GDM receive dietary recommendations on frequently consuming small meals of a low glycemic index, which would result in pregnant women with GDM appearing to have healthier dietary habits, which, in our study, were hypothesized to ameliorate glucose metabolism. Therefore, recall bias is expected to have biased our results towards the null hypothesis, and our effect estimates are expected to be closer to unity than they would be if dietary habits were assessed during the first or even second trimester of pregnancy. Furthermore, the lack of quantitative data is a limitation of this study, since our food frequency data were either semi-quantitative or qualitative.

Lastly, our adjusted logistic regression models might be affected by over-adjustment, in which case, all effect estimates might be biased towards the null hypothesis, which is that no association between dietary habits during pregnancy and GDM exists. On one hand, pre-pregnancy BMI is naturally associated with dietary habits before pregnancy, which, in turn, also affect the dietary habits during pregnancy, leading to BMI serving as a mediator of effect. The same holds true for gestational weight gain, which is influenced by dietary habits during pregnancy. To provide an explanatory example, a protective effect of a specific food category can be mediated by lower total calorie intake, as is the case with high fiber intake, which reduces appetite and can affect GDM risk via lower gestational weight gain [20]. By adjusting for pre-pregnancy BMI and gestational weight gain, that effect would be underestimated. In that sense, including pre-pregnancy BMI and gestational weight gain in our adjusted models might bias our results towards the null hypothesis. This increases the probability of a type II error (the probability of failing to reject a null hypothesis that is actually false in the population) [45]. On the other hand, pre-pregnancy BMI is a known risk factor for GDM, and its effect is not mediated solely by dietary habits but also by adiposity and its endocrine functions. In that sense, not adjusting for pre-pregnancy BMI would not account for the bias due to the effect of adiposity on the risk of GDM. This would increase the probability of a type I error (the probability of rejecting a null hypothesis that is actually true in the population) [45]. Despite the aforementioned issue with using BMI-related variables as covariates in statistical models applied in nutritional epidemiology studies, BMI before pregnancy and gestational weight gain are frequently used as covariates in nutritional epidemiological studies for GDM, since adiposity is a well-known confounder. In our study, we might have underestimated the effect of dietary habits during pregnancy on the risk of GDM by adjusting for pre-pregnancy BMI and gestational weight gain. Nonetheless, this was considered preferable over reporting results that could, in reality, reflect a type II error rather than a true association in our population.

## 5. Conclusions

In this study, we examined the associations of dietary habits during pregnancy with GDM risk. Among the examined food categories, which were chosen based on previous research findings, we observed no association of carbohydrate-rich meals, sweets, soft drinks, coffee, rice, pasta, bread and crackers, potatoes, lentils, and juices with GDM diagnosis. Cereals, fruits, and vegetables appeared to have a protective effect against GDM, while frequent tea consumption was linked to a higher risk of GDM development. Our results strengthen the associations that have been identified in previously published studies, and underline the importance and potential impact of changing dietary habits during pregnancy in adjusting one’s risk of metabolic pregnancy complications, such as GDM. Overall, the importance of healthy dietary habits prior to and during pregnancy is highlighted, with the goal of informing obstetric care specialists about offering systematic nutrition recommendations to pregnant women.

## Figures and Tables

**Table 1:** Maternal and neonatal baseline characteristics for total study population and per GDM status.

		All Study Participants	Number of Study Participants with Missing Data	Study Participants without GDM	Study Participants with GDM	
		Mean (SD)/N (%)	N (%)	Mean (SD)/N (%)	Mean (SD)/N (%)	*p*-Value ^1^
Maternal age (mean (SD))		31.53 (4.43)	0 (0.0%)	31.45 (4.43)	32.75 (4.54)	0.39
Pre-gestational BMI (mean (SD))		22.81 (3.75)	0 (0.0%)	22.70 (3.63)	24.39 (5.08)	0.20
Gestational weight gain (mean (SD))		13.46 (6.94)	0 (0.0%)	13.69 (6.63)	10.00 (10.32)	<0.01
Weight gain below IOM target range n (%)		61 (31.6%)	0 (0.0%)	54 (29.8%)	7 (58.3%)	0.08
Weight gain within IOM target range n (%)		65 (33.7%)	0 (0.0%)	61 (33.7%)	4 (33.3%)	1.00
Weight gain above IOM target range n (%)		67 (34.7%)	0 (0.0%)	66 (36.5%)	1 (8.3%)	0.10
Gravidity status n (%)	0	124 (64.2%)	0 (0.0%)	114 (63.0%)	10 (83.3%)	0.03
	1	43 (22.3%)		43 (23.8%)	0 (0.0%)	
	2	18 (9.3%)		17 (9.4%)	1 (8.3%)	
	3	6 (3.1%)		6 (3.3%)	0 (0.0%)	
	4	2 (1.0%)		1 (0.6%)	1 (8.3%)	
Parity status n (%)	0	143 (74.1%)	0 (0.0%)	133 (73.5%)	10 (83.3%)	0.22
	1	45 (23.3%)		44 (24.3%)	1 (8.3%)	
	2	5 (2.6%)		4 (2.2%)	1 (8.3%)	
Number of prior vaginal births n (%)	0	155 (80.3%)	0 (0.0%)	145 (80.1%)	10 (83.3%)	0.21
	1	34 (17.6%)		33 (18.2%)	1 (8.3%)	
	2	4 (2.1%)		3 (1.7%)	1 (8.3%)	
Number of prior caesarian sections n (%)	0	174 (90.2%)	0 (0.0%)	162 (89.5%)	12 (100.0%)	0.50
	1	19 (9.8%)		19 (10.5%)	0 (0.0%)	
Smoking before pregnancy n (%)	No	108 (56.0%)	0 (0.0%)	103 (56.9%)	5 (41.7%)	0.47
	Yes	85 (44.0%)		78 (43.1%)	7 (58.3%)	
Smoking during pregnancy n (%)	No	170 (88.1%)	0 (0.0%)	158 (87.3%)	12 (100.0%)	0.39
	Yes	23 (11.9%)		23 (12.7%)	0 (0.0%)	
Passive smoking at home during pregnancy n (%)	No	105 (54.4%)	0 (0.0%)	97 (53.6%)	8 (66.7%)	0.56
	Yes	88 (45.6%)		84 (46.4%)	4 (33.3%)	
Gestational diabetes mellitus n (%)		12 (6.2%)	0 (0.0%)	0 (0.0%)	12 (100.0%)	NA
Pregnancy-induced hypertension n (%)		7 (3.6%)	0 (0.0%)	5 (2.8%)	2 (16.7%)	0.09
Gestational age at birth (mean (SD))		38.53 (1.38)	0 (0.0%)	38.54 (1.40)	38.42 (1.16)	0.56
Birthweight in gr (mean (SD))		3075.12 (499.66)	26 (13.5%)	3060.73 (500.51)	3327.78 (433.84)	0.12
Neonate’s sex n (%)	Female	88 (52.4%)	25 (13.0%)	82 (51.6%)	6 (66.7%)	0.59
	Male	80 (47.6%)		77 (48.4%)	3 (33.3%)	
Sample Size		193		181	12	

Abbreviations: SD, standard deviation; NA, not applicable. ^1^ *p*-values are derived from the comparison of study participants with and without GDM.

**Table 2:** Crude and adjusted ^1^ results from the analysis of associations of semi-quantitative food frequency data with GDM.

	Crude *p*-Value	Adjusted *p*-Value
Carbohydrate-rich meal consumption frequency	0.22	0.26
Fruit and vegetable consumption frequency	0.07	0.04
Sweet consumption frequency	0.21	0.33
Soft drink consumption frequency	0.67	0.75
Coffee consumption frequency	0.43	0.33

^1^ In the adjusted models, maternal age, body mass index (BMI) before pregnancy, and gestational weight gain were used as covariates.

**Table 3:** Crude and adjusted ^1^ results from the analysis of associations of qualitative food frequency data with GDM.

	Crude Analysis		Adjusted Analysis	
	Odds Ratio (OR)	*p*-Value	Odds Ratio (OR)	*p*-Value
Frequent consumption of cereals	0.30	0.045	0.35	0.095
Frequent consumption of rice	0.60	0.39	0.70	0.57
Frequent consumption of pasta	0.64	0.53	0.66	0.56
Frequent consumption of bread and crackers	1.52	0.54	1.55	0.53
Frequent consumption of potatoes	1.24	0.75	1.30	0.71
Frequent consumption of lentils	2.48	0.25	2.50	0.25
Frequent consumption of tea	3.09	0.067	3.97	0.035
Frequent consumption of juices	0.50	0.26	0.56	0.35

^1^ In the adjusted models, maternal age, body mass index (BMI) before pregnancy, and gestational weight gain were used as covariates.

## Data Availability

The data analysed in this study can be made available upon request. The data are not publicly available due to privacy regulations.

## Supplementary Materials

The following supporting information can be downloaded at: https://www.mdpi.com/article/10.3390/jcm12051857/s1, Table S1: Maternal dietary habits during pregnancy; Table S2: Odds ratios and 95% confidence intervals from crude and adjusted logistic regression analyses for fruit and vegetable consumption during pregnancy.
