# Supplementary material for: The Role of Diet during Pregnancy in Protecting against Gestational Diabetes Mellitus in a Population with Mediterranean Dietary Habits: A Cross-Sectional Study
Source: J Clin Med. 2023 Feb 26;12(5):1857. doi: 10.3390/jcm12051857 (PMC10003761; doi:10.3390/jcm12051857)
Supplement: Supplementary file 1 [file jcm-12-01857-s001.zip › jcm-2178062-supplementary.pdf]

Table S1. Maternal dietary habits during pregnancy

		N(%)	
**Semi-quantitative Data**			
Daily frequency of carbohydrates-rich meals consumption n(%)	None	2 (1.0)	
	1 time / day	53 (27.5)	
	2 times / day	86 (44.6)	
	3 times / day	41 (21.2)	
	4 times / day	9 (4.7)	
	5 times / day	2 (1.0)	
Frequency of sweets consumption n(%)	None	18 (9.3)	
	1 time / week	29 (15.0)	
	2 times / week	37 (19.2)	
	3-4 times / week	54 (28.0)	
	1 time / day	36 (18.7)	
	2 times / day	19 (9.8)	
Daily frequency of fruits and vegetables consumption n(%)	None	3 (1.6)	
	1 time / day	33 (17.1)	
	2 times / day	74 (38.3)	
	3 times / day	45 (23.3)	
	4 times / day	28 (14.5)	
	5 times / day	10 (5.2)	
Frequency of soft drinks consumption n(%)	None	136 (70.8)	
	3 times / week	33 (17.2)	
	1 time / day	20 (10.4)	
	2-3 times / day	3 (1.6)	
Frequency of coffee consumption n(%)	None	77 (39.9)	
	3 times / week	27 (14.0)	
	1 time / day	76 (39.4)	
	2-3 times / day	11 (5.7)	
	4-5 times / day	2 (1.0)	
**Qualitative Data**			
Frequent consumption of cereals n(%)	No	60 (31.1)	
	Yes	133 (68.9)	
Frequent consumption of rice n(%)	No	59 (30.6)	
	Yes	134 (69.4)	
Frequent consumption of pasta n(%)	No	35 (18.1)	
	Yes	158 (81.9)	
Frequent consumption of bread and crackers n(%)	No	64 (33.2)	
	Yes	129 (66.8)	
Frequent consumption of potatoes n(%)	No	56 (29.0)	

	Yes	137 (71.0)	
Frequent consumption of lentils n(%)	No	62 (32.1)	
	Yes	131 (67.9)	
Frequent consumption of tea n(%)	No	154 (79.8)	
	Yes	39 (20.2)	
Frequent consumption of juices n(%)	No	53 (27.5)	
	Yes	140 (72.5)	
Total Sample Size		193	

Table S2. Odds ratios and 95% confidence intevals from crude and adjusted logistic regression analyses for fruits and vegetables consumption during pregnancy

	Crude Analysis			
		Odds Ratio	95% confidence intervals	
Daily frequency of fruits and vegetables consumption	None	Reference	-	-
	1 time / day	0,13	0,01	2,11
	2 times / day	0,08	0,01	1,21
	3 times / day	0,05	0,00	1,02
	4 times / day	0,43	0,03	5,78
	5 times / day	0,00	0,00	Infinity
	Adjusted Analysis			
Daily frequency of fruits and vegetables consumption	None	Reference	-	-
	1 time / day	0,03	0,00	0,90
	2 times / day	0,02	0,00	0,46
	3 times / day	0,01	0,00	0,47
	4 times / day	0,11	0,00	2,66
	5 times / day	0,00	0,00	Infinity
